# A new era in maternal–child health: Abrysvo's role in RSV prevention

**DOI:** 10.1002/hsr2.2236

**Published:** 2024-07-03

**Authors:** Ayesha Shaukat, Um E. Abiha Batool, Nathalie Nasser

**Affiliations:** ^1^ Department of Internal Medicine Dow University of Health Sciences Karachi Pakistan; ^2^ Faculty of Medical Sciences Lebanese University Beirut Lebanon

To the Editor,

In a significant stride forward in the realm of maternal and neonatal healthcare, the US Food and Drug Administration (FDA) has approved *Abrysvo* (respiratory syncytial virus vaccine), representing a historic breakthrough that ushers in the era of the first‐ever vaccine specifically designed for pregnant individuals.^1^ *Abrysvo* is a shining beacon of preventive medicine, engineered to safeguard against lower respiratory tract disease (LRTD), including the notably severe form attributed to respiratory syncytial virus (RSV) in neonates.^1^ The gravity of RSV infections poses a formidable threat to infant well‐being, a threat further magnified when infants are born prematurely or have underlying health conditions.^1^ Remarkably, this historic decision builds upon the milestone established last year when the FDA, on May 2023, accorded *Abrysvo* its approval for shielding individuals aged 60 and above against LRTD precipitated by RSV.^1^ Notably, the most severe cases and highest hospitalization rates are prevalent in infants aged less than 5 months.^2^ RSV exerts its impact on a significant majority of children, with approximately 60%–70% experiencing its effects in their first year and nearly all children being affected by the age of 2.^3^ Furthermore, RSV is the primary causative agent in 45%–90% of bronchiolitis cases and 15%–35% of pneumonia cases.^3^ Beyond its respiratory effects, RSV is gaining recognition for its extrapulmonary manifestations, particularly neurological involvement.

A seasonal disease by nature, RSV stands as one of the leading causes of severe acute lower respiratory Infection (ALRI) and mild upper respiratory Infection in infants and young children. The vulnerability to RSV infection is most pronounced in specific groups, including premature infants, those with neuromuscular issues, individuals with weakened immune systems, and those with underlying lung or heart conditions.^3^ The modes of RSV transmission encompasses airborne transmission via coughs or sneezes, direct contact such as touching or handshakes, contact with virus‐contaminated surfaces, and exposure to virus‐laden droplets.^3^

Two poignant examples highlight the severe consequences of RSV infection, both in infants and pregnant individuals with underlying respiratory conditions. Bottino et al. illustrated the devastating impact of RSV in their report, which focused on a 40‐day‐old male infant.^3^ Tragically, this infant's condition rapidly deteriorated as a result of an RSV infection, leading to the development of cerebral edema and, ultimately, the loss of life.^3^ Moreover, Wheeler et al. shed light on the alarming concerns surrounding severe maternal complications linked to RSV infections, particularly in cases where mothers have pre‐existing lung conditions like asthma.^4^ Their comprehensive study disclosed that among the three patients examined, two necessitated intensive care unit (ICU) level care and intubation due to the severity of their RSV‐related complications.^4^ They underscore the potential gravity of RSV‐related complications and the imperative for early intervention and specialized care to mitigate adverse outcomes.

The risk of infant mortality due to RSV infection, while less common in resource‐abundant settings, remains prevalent in underdeveloped areas.^2^ Severe RSV infection during infancy has been associated with recurrent wheezing and asthma in later childhood.^2^ The creation of an RSV‐targeted vaccination is essential for the possibility of universal infant protection. Yet, because acute RSV disease often occurs within the first 3–4 months of life, the essential window for active immunization to induce protection is constrained.^2^ The placental transfer of maternal RSV‐specific IgG generated by the vaccination offers one therapeutic remedy.^2^ This provides a significant benefit by bridging the interval between birth and the time the infant's immune system matures enough to mount a successful fight against RSV.^2^

While monoclonal antibodies (mAb) such as Nirsevimab and Palivizumab effectively protects against RSV infection in newborns and infants, but its use is constrained by certain limitations.^2^ These antibodies are typically reserved for at‐risk infants, including preterm infants, those during the high RSV season, or those with underlying comorbidities.^2^ While it effectively safeguards vulnerable infants, many otherwise healthy infants who may still be at risk of RSV infection do not receive the protective benefits of these antibodies.

The approval of *Abrysvo* is grounded in the findings of a Phase III randomized double‐blinded, placebo‐controlled clinical trial.^5^ This trial involves assessing approximately 3500 pregnant individuals at various gestational stages ranging from 24 to 36 weeks receiving vaccines as part of the study.^5^ Participants received a single intramuscular injection of 120 μg of a bivalent RSV prefusion F protein–based vaccine (RSVpreF), while an equivalent number of participants received a placebo.^5^ The results demonstrated a remarkable 81.8 percent reduction in the risk of severe lower respiratory tract disease (LRTD) within the initial 90 days following birth. The efficacy remained substantial, with a risk reduction rate of 69.4% within the first 180 days after birth without significant adverse effects.^5^ The ABRYSVO vaccine represents the sole RSV immunization administered to expectant mothers during the 32nd to 36th weeks of pregnancy, aiming to safeguard infants from birth up to 6 months of age.^5^

The FDA approval of the groundbreaking *Abrysvo* for pregnant individuals against RSV marks a significant stride forward in maternal–child health. This milestone alleviates pressing health concerns, offering the promise of healthier beginnings for infants and easing parents' anxieties. This innovative approach, targeting maternal immunity to safeguard infants and mothers, emphasizes maternal well‐being's pivotal role in infant health. This approval catalyzes future advancements in RSV prevention and maternal–child health.

## AUTHOR CONTRIBUTIONS

**Ayesha Shaukat**: Conceptualization; writing—original draft. **Nathalie Nasser**: Writing—review & editing.

## CONFLICT OF INTEREST STATEMENT

The authors declare no conflicts of interest.

## ETHICS STATEMENT

Ethics approval from IRB is not required.

## TRANSPARENCY STATEMENT

The lead author Ayesha Shaukat affirms that this manuscript is an honest, accurate, and transparent account of the study being reported; that no important aspects of the study have been omitted; and that any discrepancies from the study as planned (and, if relevant, registered) have been explained. All authors have read and approved the final version of the manuscript, corresponding author Nathalie Nasser had full access to all of the data in this study and takes complete responsibility for the integrity of the data and the accuracy of the data analysis.

## Data Availability

The authors have nothing to report. Data sharing is not applicable to this article as no datasets were generated or analyzed during the current study.
